# Degenerative Meniscus in Knee Osteoarthritis: From Pathology to Treatment

**DOI:** 10.3390/life12040603

**Published:** 2022-04-18

**Authors:** Nobutake Ozeki, Hideyuki Koga, Ichiro Sekiya

**Affiliations:** 1 Center for Stem Cell and Regenerative Medicine, Tokyo Medical and Dental University (TMDU), 1-5-45 Yushima, Bunkyo-ku, Tokyo 113-8519, Japan; sekiya.arm@tmd.ac.jp; 2 Department of Joint Surgery and Sports Medicine, Graduate School of Medical and Dental Sciences, Tokyo Medical and Dental University (TMDU), 1-5-45 Yushima, Bunkyo-ku, Tokyo 113-8519, Japan; koga.orj@tmd.ac.jp

**Keywords:** meniscus, osteoarthritis, medial meniscal extrusion, medial meniscus posterior root tear, meniscal repair, centralization

## Abstract

Knee osteoarthritis is a common degenerative joint disease characterized by chronic knee pain and disability in daily living. The lesion can involve the cartilage as well as the synovium, bone, ligaments, and meniscus, indicating a complicated pathology for knee osteoarthritis. The association with the meniscus has recently attracted much attention. Meniscal tears can initiate and progress knee osteoarthritis, with deleterious effects on the important roles of the meniscus in load distribution, shock absorption, and stability of the knee joint. Degenerative meniscus lesions are commonly observed in elderly people, but they have less impact on the prognosis of osteoarthritis. However, they are often accompanied by meniscal extrusion, which substantially decreases the hoop function of the meniscus and increases the risk of knee osteoarthritis. When surgical treatment is necessary, meniscal tears should be repaired to the greatest extent possible to preserve meniscus function. Long-term studies show better clinical outcomes and less degenerative osteoarthritis changes following meniscal repair than following partial meniscectomy. For meniscal extrusion repair, centralization techniques have been proposed that involve suturing the meniscus-capsule complex to the edge of the tibial plateau. Advancements in orthobiologics, such as platelet-rich plasma or stem cell therapy, have the potential to prevent the initiation or progression of osteoarthritis.

## 1. Introduction

Knee osteoarthritis (OA), one of the most prevalent degenerative musculoskeletal diseases, has several known risk factors, including old age, female sex, malalignment, obesity, genetic factors, and trauma [1,2]. The number of knee OA or primary knee replacement surgeries is increasing globally due to aging populations [3,4]; therefore, treatments and preventive measures based on a better understanding of the etiology/pathology of knee OA are needed. However, no effective pharmaceutical therapy is clinically available due to the complicated pathology of knee OA, as this disorder affects the structures of the whole knee joint, including the articular cartilage, subchondral bone, synovium, ligament, and meniscus [5,6]. Recently, the meniscus has been a focus of the studies on the initiation/progression of knee OA [7].

**Figure 1:**
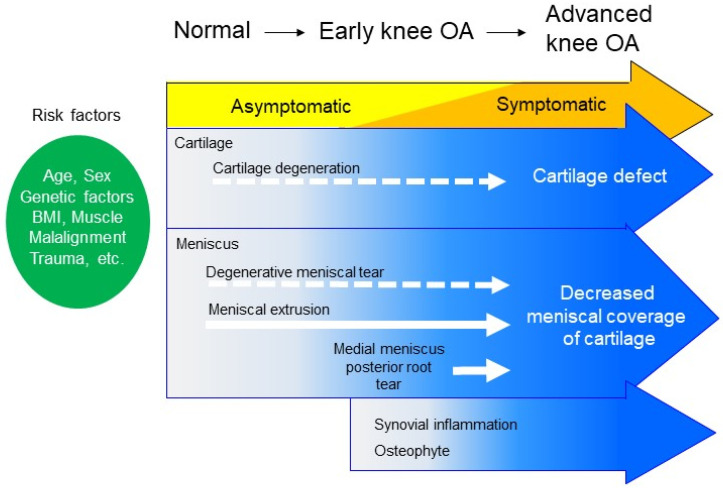
Scheme of knee osteoarthritis (OA) progression. In knee OA, cartilage degeneration progresses gradually based on several risk factors, including age, sex, genetic factors, bone mass index, muscle, alignment, and trauma [6]. Knee symptoms gradually progress with repeated improvement and exacerbation, and early knee OA progresses to advanced knee OA. Degenerative meniscal tears, which are typically observed in elderly people, also progress gradually, but they do not substantially affect the prognosis of OA. By contrast, meniscal extrusion, especially when followed by a medial meniscus posterior root tear, is a critical factor for the initiation/progression of knee OA, as it results in decreased meniscus coverage of the cartilage [16]. This is a quite different pathology from a simple degenerative meniscal tear [17]. Along with OA progression, synovial inflammation and osteophyte formation are commonly observed. OA; osteoarthritis; BMI; body mass index.

Meniscectomy is used to treat meniscus tears, but it is well-known to decrease meniscal function and is often followed by the initiation and development of knee OA during long-term follow-up [8,9]. This indicates a critical role for the meniscus in the initiation/progression of OA. However, a degenerative tear of the meniscus, if not significant, does not always induce significant knee symptoms, such as pain, aching, or stiffness [10], so meniscal surgery is not indicated for all degenerative meniscal tears [11,12,13]. Nevertheless, recent magnetic resonance imaging (MRI) studies have revealed that the meniscus has the variety of pathologies that dramatically affect the hoop function of the meniscus and tend to initiate knee OA [14,15]. A representative case is meniscal extrusion, especially when accompanied by a meniscus root tear, which is quite different from a simple degenerative tear (Figure 1). The patients are usually middle-aged or elderly people, and acute severe pain occurs after a minor trauma, such as occurs by stumbling in a step. Failure to attend to these pathologies when diagnosed by MRI or ultrasound can result in the loss of the window of the opportunity to prevent the progression to OA.

In this review article, we present the critical pathologies of the meniscus involved in knee OA and treatments for the meniscus that can prevent the initiation/progression of knee OA. Although knee OA is recognized as a disease of the cartilage, we speculate that the meniscus is much more involved than initially thought.

## 2. Meniscus Structures and Functions

The meniscus is a fibrocartilage located between the femoral condyle and the tibial plateau and covers more than half of the tibial plateau [18]. The roles of the meniscus are load distribution, lubrication, and stability of the knee joint [18]. These functions are characterized by major components of the meniscus, such as water, collagen, and glycosaminoglycan. The clinical association between joint space narrowing and meniscectomy indicates an essential function of the meniscus for load transmission [19]. Several laboratory studies have confirmed that total removal of the meniscus results in a 40–50% decreased contact area of the compartment and a two- to three-fold increase in peak contact stresses compared to the intact knee, and this may contribute to increasing stress concentrated on both the articular cartilage and the subchondral bone [20]. Axial forces loaded onto the meniscus during weight bearing are converted into tensile strain through the circumferential collagen fibers of the meniscus, expressed as its hoop function [18]. The attachment sites of the anterior and posterior meniscus roots are critical to this function, as they stabilize the meniscus to the tibia.

The cells of the meniscus differ according to their location; the outer zone cells are classified as fibroblasts, and the inner zone cells are similar to fibrochondrocytes [18]. Vascularization is limited to the peripheral 10–30% of the meniscus, so avascular lesions of the meniscus have poor healing potential even after meniscal repair [21].

## 3. Meniscus Pathology and Knee OA

### 3.1. Traumatic Meniscal Tears 

Meniscal tears can be caused by traumatic knee injury, or they can arise due to degenerative changes without traumatic episodes. The ESSKA (European Society of Sports Traumatology, Knee Surgery, Arthroscopy) European meniscus consensus group has defined traumatic meniscus injury as a meniscal tear that is associated with sufficient knee injury and a sudden onset of knee pain [13]. The natural course of traumatic meniscus injury in adolescents or in association with sports injuries has not been elucidated due to a lack of solid evidence. Traumatic meniscal tears occur in isolation but are commonly detected in conjunction with ligament injuries, especially anterior cruciate ligament (ACL) injury. In a cohort of ACL injuries, 820 patients (58.7%) of the 1398 ACL injuries had associated meniscal tears at the time of injury [22]. 

**Figure 2:**
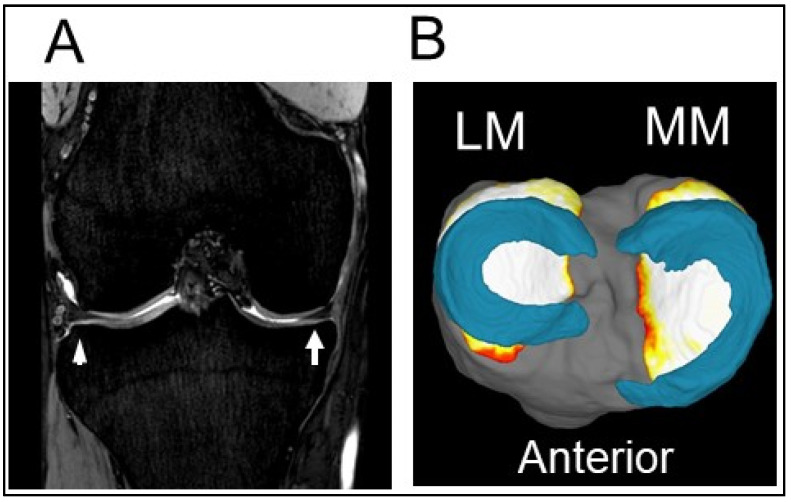
A normal meniscus in the right knee. (**A**) Coronal view of the normal meniscus. Arrow indicates the MM. Arrowhead indicates the LM. (**B**) A 3D MRI view reconstructed using SYNAPSE 3D software (Japanese product name: SYNAPSE VINCENT; FUJIFILM Corporation, Tokyo, Japan). MM, medial meniscus; LM, lateral meniscus; MTP, medial tibial plateau.

Morphological tear patterns of the meniscus can include longitudinal, radial, horizontal, and flap tears [14]. MRI is a useful method for the evaluation of meniscus (Figure 2) and has a diagnostic accuracy value of up to 90% [23,24,25]. From the viewpoint of the hoop function of the meniscus, radial tears that transect the meniscus from the inner free edge to the periphery result in substantial loss of the load distribution [26]. Similarly, posterior root tears severely affect the biomechanics of the knee joint. A lateral meniscus (LM) posterior root tear is often accompanied by an acute ACL injury [27,28], while a medial meniscus posterior root tear (MMPRT) typically occurs in middle-aged and elderly patients, appearing as acute severe pain after a minor trauma, such as stumbling in a step. An MMPRT dramatically increases the contact pressure on the medial tibial plateau in a manner equivalent to a complete meniscectomy [29] and may induce subchondral insufficiency fractures or bone marrow edema [30,31]. This pathology has been recognized as a strong risk factor for the progression of knee OA, as discussed in a later section. 

### 3.2. Degenerative Meniscus Lesions

Degenerative meniscus lesions (DMLs) are defined as meniscus lesions that occur without a history of knee trauma in patients older than 35 years [13]. DMLs develop slowly and typically involve a horizontal cleavage [32]. MRI can identify a linear intra-meniscus signal communicating with the articular surface in elderly people; this phenomenon is considered a degenerative process. One MRI analysis reported meniscal tears in 35% of persons older than 50 years of age; however, two-thirds of these tears were asymptomatic [10]. The histology of torn menisci at the time of arthroscopic meniscectomy indicates that meniscal tears in patients older than 40 years are associated with decreased cellularity of the meniscus [33]. A human fresh cadaver study showed that the menisci in older patients had a decreased cell density or diffused hyper cellularity, along with cellular hypertrophy and abnormal cell clusters [34]. These findings indicate that the degenerated meniscus is more vulnerable to injury. A multicenter study showed that elderly people who showed no baseline radiographic OA but who demonstrated OA progression during a 30-month follow-up period had significantly more meniscus injuries evident in the baseline MRI compared to controls who showed no progression of OA [35]. A 10-year cohort study showed that middle-aged people who had an medial meniscus (MM) injury apparent in a baseline MRI showed a significantly progressed radiographic OA compared with people without a meniscus injury [36]. These studies indicate that a DML is highly correlated with the initiation of OA. Conversely, the rate of MM lesions (tears or degeneration) was not significantly higher in patients who developed incident OA compared with control patients, although MM extrusion was more common at baseline in the cases compared with the controls [37]. Katz et al. [38,39] conducted a multicenter RCT involving symptomatic patients with mild-to-moderate OA and degenerative meniscal tears to compare functional outcomes following arthroscopic partial meniscectomy (APM) versus physiotherapy (PT). Over a 5-year follow-up, pain improved considerably in both groups; however, TKAs were performed in 1.8% of the PT group and 9.8% of the APM group, and the ratio of TKA was greater among those who were randomized to undergo APM [39]. The relationship between DML and OA remains controversial (Figure 1).

### 3.3. Meniscal Extrusion

**Figure 3:**
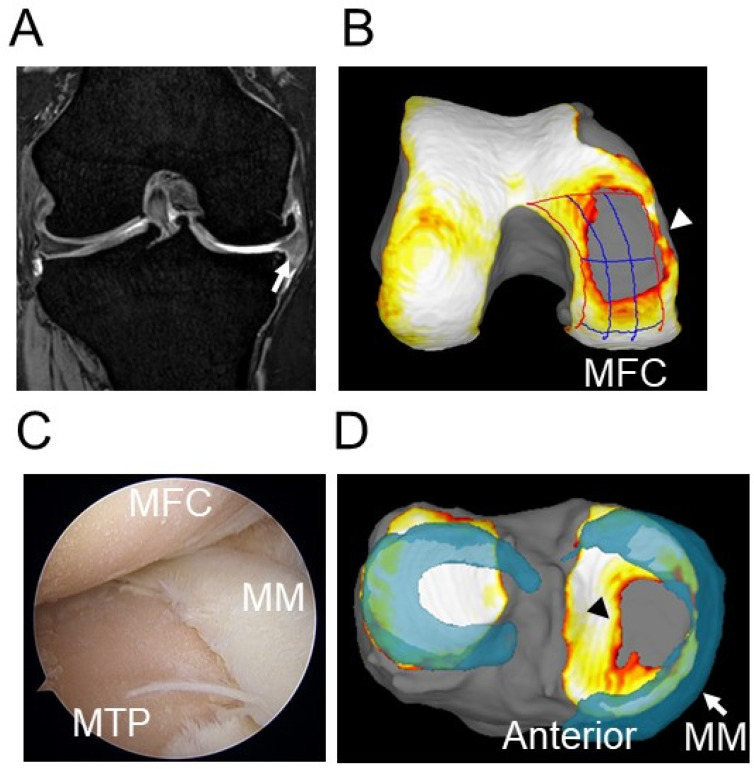
An extruded medial meniscus in the right knee. (**A**) Medial meniscal extrusion was confirmed in a coronal view (white arrow). Cartilage defects were observed in the medial compartment. (**B**) The 3D MRI view shows the cartilage defect in the MFC (white arrowhead). (**C**) Arthroscopic findings indicate decreased meniscal coverage of the MTP and cartilage defects in the MFC and the MTP. (**D**) The 3D MRI view shows extrusion of the MM (white arrow) and a cartilage defect in the MTP (black arrowhead). MM, medial meniscus; LM, lateral meniscus; MFC, medial femoral condyle; MTP, medial tibial plateau.

Meniscal extrusion is a displacement of the meniscus body beyond the outermost margin of the tibial plateau [40] and is often accompanied by OA (Figure 3), a radial tear, or a posterior root tear of the meniscus. Hada et al. reported that medial tibial osteophytes were frequently detected by MRI in patients with early stage knee OA, indicating a close association with MM extrusion (MME) [41]. Conversely, Krych et al. reported that disruption of the meniscotibial ligament, which connects the inferior edges of the meniscus to the periphery of the tibial plateau, induced meniscal extrusion [42].

Meniscal extrusion causes a decreased coverage of the tibial plateau, thereby leading to an increased load bearing of the cartilage. Therefore, meniscal extrusion is more strongly correlated with joint space narrowing when compared to meniscal tears and cartilage defects [43,44], and meniscal extrusion is an independent predictor of OA disease progression [45,46]. The database of the Knee Osteoarthritis Initiative indicated that the rate of MM tearing or degeneration was not significantly higher in those who developed incident OA than in control patients; however, MME was more common at baseline in the cases than in the controls [37]. In addition, knee pain was more frequently seen in OA patients with MME than in those without MME [47,48].

MMPRT is a strong cause of MME and results in the loss of the meniscus hoop function, while also accelerating progression of the degenerative changes of knee OA (Figure 1). The biomechanics of MMPRT are similar to those observed following total meniscectomy; consequently, MMPRT is a strong risk factor for knee OA [29]. A retrospective review of 52 patients with symptomatic MMPRT showed that 16 patients (31%) who underwent total knee arthroplasty at a mean of 30 months after diagnosis [16] had an overall failure rate, based on clinical and radiographic criteria, of 87% following non-operative treatment. 

The widely accepted threshold of MME is a displacement of the meniscus body of more than 3 mm [49]. However, the amount of displacement that qualifies as a pathologic change has not been established. Some scoring systems have been described for 2D coronal MRI data: Grade 0 = no extrusion; Grade 1 = partial extrusion; and Grade 2 = complete extrusion without any contact with the joint space [50]. MME can also be scored as a relative percentage of extrusion, with Grade 0 = No extrusion; Grade 1 = ≤ 50% extrusion; and Grade 2 ≥ 50% extrusion [51,52]. Similarly, it can be scored as part of the semi-quantitative MRI scoring systems for OA, with Grade 0 = <2 mm; Grade 1 = 2–2.9 mm, Grade 2 = 3–4.9 mm; and Grade 3 = > 5 mm [53]. The use of 3D MRI has also been assessed for quantification of meniscal extrusion [54]. For example, Sharma et al. used 3D MRI measurements to investigate the association of the meniscus position and size with the subsequent structural progression of medial knee OA [55]. Their findings showed that the MME obtained from the central slice of the MM was significantly greater in knees with subsequent OA progression than in knees without subsequent progression, although no significant difference was observed in most measures of the MM morphology. Fully automated segmentation to reconstruct the 3D morphology of knee cartilage and meniscus has been recently established and can evaluate meniscal extrusion by the meniscal extrusion width or volume and by assessing the percentage of meniscus coverage of the tibial plateau.

Ultrasound is also useful for detecting meniscal extrusion, and it can establish the effect of the weight bearing position in MME [56,57]. MME is considered a typical pathology of OA and an established risk factor of initiation/progression of OA (Figure 1); therefore, further elucidation of MME will lead to new interventions for the prevention of OA.

## 4. Treatment for Degenerative Meniscal Lesions

### 4.1. Meniscectomy versus Conservative Treatment

Meniscectomy is well-known to lead to radiographic and symptomatic OA in the long term [58]. Fairbank was the first to describe the radiological changes occurring after meniscectomy [19]. Pengas et al. conducted a mean of 40 years (33 to 50) follow-up after total meniscectomy and found an incidence of knee OA of 81%, according to the Kellgren and Lawrence grade, whereas non-operated knees had an incidence of only 18% [59]. Overall, 13% had already undergone TKA at the time of follow-up, and those patients showed a 132-fold increase in the rate of TKA in comparison to their geographical and age-matched peers. A 16-year follow-up study of partial meniscectomy for isolated meniscal tears showed that meniscectomy induced a high risk of both radiographic and symptomatic femorotibial OA compared with matched controls, even if the amount of meniscal resection was limited [60]. Factors that were correlated with worse outcomes included degenerative meniscal tears and extensive resections. These studies indicated that meniscectomy leads to symptomatic OA later in life, even after a small resection of the meniscus. Nevertheless, partial meniscectomy remains a popular surgical procedure, especially for symptomatic DML [61]. 

Randomized clinical trials that compared APM and PT for symptomatic DML showed no significant differences in patient objective outcomes at short-term or mid-term follow-up [38,62]. A multicenter, randomized, double-blind, sham-controlled trial showed that the outcomes after APM were no better than those after a sham surgical procedure [11]. These results suggest that unnecessary meniscectomies should be avoided, especially in cases with DML. The ESSKA consensus group proposed that APM in DML should only be considered after a proper standardized clinical and radiological evaluation and when the response to non-operative management has not been satisfactory [13].

In regard to traumatic meniscal tears, the prevalence rate of developing moderate to severe OA was 29% after ACL reconstruction in a minimum 20-year follow-up study, and the odds ratios for statistically significant factors for the presence of OA were 3.0 for medial meniscectomy [63]. The evidence for OA progression is not sufficient for traumatic meniscal tears; however, meniscectomy will induce OA changes similar to those seen in DML cases in the long-term follow-up.

### 4.2. Meniscus Preservation Surgery 

When surgical treatment is necessary for meniscal tears, the first option should be meniscal repair to preserve the meniscus function [15]. The ratio of meniscal repairs to meniscectomy has increased gradually with the development of the concept to preserve the meniscus [61]. Meniscal repair has been performed for longitudinal tears within the vascular area of the meniscus. Although a higher reoperation rate was reported for meniscal repair than for meniscectomy, fewer degenerative radiographic changes were observed in meniscal repair than with APM after more than 10 years of follow-up [64]. A retrospective study conducted for more than 10 years showed that meniscal repair provided a better functional score than APM, and the radiological scores also correlated closely [65]. These findings support the idea that meniscal repair may protect against OA progression during long-term follow-up, although further prospective randomized studies are necessary for confirmation. Based on these findings, the indications for meniscal repair have been expanding along with improvements in meniscal repair techniques and orthobiologics.

In the case of acute MMPRT, surgical repair should be considered more necessary than for other meniscus injuries due to the high risk of progression of cartilage degeneration. Therefore, accurate diagnosis by MRI and meeting the window of opportunity for treatment are quite crucial [66]. Meniscus root repair is often performed by placing sutures through the meniscus root and pulling them through a transtibial tunnel to reattach the root to the tibia. High survival clinical rates of 99% at 5 years and 95% at 7 years were reported when failure was defined as conversion to TKA or a poor final Lysholm score [67]. Even though good healing status and clinical results were obtained, meniscal extrusion remained in most cases [68]. Augmentation of the centralization technique will reduce this meniscal extrusion and preserve the meniscus function over a longer period [69]. A systematic review comparing 355 patients with MMPRT who underwent either meniscal repair (n = 229), meniscectomy (n = 74), or non-operative treatment (n = 41) reported incidences of OA development of 53%, 99%, and 95%, respectively, and total knee arthroplasty (TKA) rates of 34%, 52%, and 46%, respectively [70]. The discounted costs over 10 years were $22,590 for meniscal repair, as opposed to $31,528 and $25,006 for meniscectomy and non-operative treatment, respectively. Therefore, to prevent the progression of OA, MMPRT repair should be considered early, when possible. For active elderly people, MMPRT repair accompanied by high tibial osteotomy (HTO) is commonly indicated for this pathology.

Meniscal centralization has recently been developed for the treatment of meniscal extrusion. Koga et al. first reported arthroscopic centralization of an extruded LM by suturing the capsule attached to the meniscus to the edge of the tibial plateau using suture anchors [71]. Satisfactory clinical results and a significant reduction in meniscal extrusion on MRI were obtained at the 2-year follow-up [72]. In addition, radiographic evaluation showed an increased joint space width for up to 2 years [72]. Centralization is also applied as an augmentation of MMPRT repair [69,73] or meniscal allograft transplantation [74]. Biomechanical studies have provided evidence of restoration of the load distributing function of the meniscus by this procedure [75,76,77]; therefore, it has the potential to prevent the progression to OA.

### 4.3. Knee Osteotomy

When malalignment of the lower leg is present as part of the meniscus pathology, preventing degenerative changes is difficult using only a meniscus preservation surgery [78]. Therefore, knee osteotomy is considered in combination with meniscus preservation surgery, and this can correct the malalignment to decrease the loading to the damaged cartilage. The most common osteotomies for varus alignment are the medial opening wedge HTO, or lateral closed wedge HTO. A systematic review showed good recovery to sports/work after the HTO [79]. In addition, the HTO, combined with meniscal centralization, showed a greater widening of the joint space compared with HTO alone [80]. Conversely, Choi, et al. [81] and Astur, et al. [82] reported that follow-up MRI showed decreased MM extrusion after HTO alone. Further studies are necessary to elucidate whether the combination of meniscus surgeries with HTO can avoid the need for arthroplasty over a longer period.

### 4.4. Meniscus Replacement 

Meniscus replacement has been clinically performed for meniscus deficiency after meniscectomy. Acceptable short- or mid-term clinical results of meniscal allograft transplantation have been reported in painful knees that had previously been subjected to subtotal or total meniscectomy [83,84]. Collagen meniscus implants composed of collagen type I scaffolds are an option for meniscus substitution. Successful clinical results and radiological findings were reported at a 10-year follow-up after collagen meniscus implant transplantations [85]. Artificial materials, such as biodegradable polyurethane, have been tested as meniscal scaffolds. Satisfactory clinical outcomes were obtained at the 5-year follow-up, even though MRI scans showed a smaller size of the scaffold compared with that of the intact meniscus [86]. 

However, approval for use of these meniscal materials differs depending on the country. Autogenous tendon is also available as a potential for meniscus substitution and has strong tensile properties [87,88]. However, meniscus replacement by an autogenous tendon graft in meniscus-deficient knees of OA patients has not been successful [89]. A recent report showed promising potential of the semitendinosus tendon as a meniscus transplant, even in early results [90]. In this series, the doubled semitendinosus tendon was transplanted through bone tunnels for root anchorage, and the graft was sutured with all-inside, inside-out, and outside-in techniques. Surgical improvements and appropriate indications would further improve the outcome of this procedure. Various other materials, such as fiber-reinforced scaffolds [91] or high-hydrostatic-pressure decellularized menisci [92], have been tested in preclinical trials, and they showed potential to function as meniscus substitutes.

### 4.5. Orthobiologics 

Recently, orthobiologics have emerged as potential materials to improve the poor healing potential of the meniscus due to its inadequate blood supply, especially in the inner margin of the meniscus [93,94]. In a cohort study with 1239 patients, the reoperation rate was significantly lower in patients who underwent a meniscal repair in conjunction with ACL reconstruction (9.7%) than in patients who underwent meniscal repair alone (16.7%) at the 2-year follow-up [95]. Another cohort study showed no significant difference in any of the postoperative outcome measures and a similar failure rate after meniscal repair in two groups: meniscal repair with a marrow venting procedure (12.9%) and meniscal repair with concomitant ACL reconstruction (7.8%) [96]. These results indicated that bone marrow stimulation procedures, in conjunction with isolated meniscal repair, have some potential to enhance healing of the repair. Biological factors released while drilling the bone tunnel may contribute to the enhanced healing potential of the meniscus. Fibrin clots derived from peripheral blood contain growth factors and act as a scaffold that enhances meniscus healing. In a series of meniscal repairs for radial tears, second-look arthroscopy showed that healing of the periphery occurred in all seven cases [97,98].

Platelet-rich plasma (PRP), which contains growth factors, including transforming growth factor-β, platelet-derived growth factor, vascular endothelial growth factor, and basic fibroblast growth factor, is clinically used in meniscal repair. However, its efficacy is still a matter of controversy due to the differences in indications or PRP preparation techniques [99]. Successful effects of PRP were reported in terms of the decreased risk of failure for isolated meniscal repair over 3 years [100]. However, PRP did not reduce the risk of meniscal repair failure in the setting of concomitant ACL reconstruction, suggesting that bone tunnel drilling provides sufficient bleeding and introduction of growth factors into the knee joint and that additional administration of PRP is unnecessary. A systematic review stated that the available evidence is still insufficient to support PRP augmentation of meniscal repair surgery to improve function or MRI evaluation compared with standard repair techniques [99]. Further evidence with a longer follow-up should be sought in the future.

Stem cells are promising orthobiologics that can change the strategy of meniscus treatment. A single center, prospective, first-in-human safety study showed that implantation of autologous bone marrow-derived mesenchymal stem cells (MSCs) in a collagen scaffold into the avascular meniscal tear prior to meniscal repair resulted in 3 of 5 patients being asymptomatic at the 2-year follow-up and showing improved clinical scores and MRI findings [101]. 

Synovial MSCs are a reasonable cell source for regenerative medicine of the meniscus due to their high proliferative and chondrogenic potential [102], and they have the same origin as intra-articular tissue [103]. Satisfactory clinical results were reported two years after transplantation of synovial MSCs onto menisci repaired for degenerative tears, and 3D MRI showed no evidence of tears at the repair site [104]. Allogenic MSCs are acceptable for use without triggering immunologic reactions. A randomized double-blind controlled study showed that patients who had injections of allogeneic bone marrow-derived MSCs after partial meniscectomy experienced a significant reduction in pain for up to 2 years compared with controls who had an injection of hyaluronic acid. The safety of allogenic MSCs was confirmed, and a significantly increased volume of meniscus in the sequential MRI was reported [105]. Conversely, the addition of MSCs with a polyurethane scaffold in meniscectomized knee did not show any advantage in the protection of articular cartilage [106]. Stem cells have significant potential to enhance healing after meniscal repair, especially in avascular zones or degenerative conditions of the meniscus. Development of regenerative medicine as a standard procedure in meniscus treatment will still require the accumulation of high-quality evidence based on RCTs.

## 5. Conclusions

The meniscus has a critical role in OA initiation/progression, especially in middle-aged and elderly people. Preservation of the function of the meniscus is quite important for preventing the progression of OA. When surgical option is necessary for a meniscal tear, the recommended procedure is meniscal repair, rather than meniscectomy. However, PT can be applied to DML without any other pathology. When MMPRT or MME is present, a surgical procedure that preserves the meniscus function should be considered. In this field, longer-term follow-up and validated evidence are still necessary to accomplish the prevention of OA.

## Abbreviations

ACL	anterior cruciate ligament
APM	arthroscopic partial meniscectomy
DML	degenerative meniscal lesions
HTO	high tibial osteotomy
LM	lateral meniscus
MM	medial meniscus
MME	medial meniscal extrusion
MMPRT	medial meniscus posterior root tear
MRI	magnetic resonance imaging
MSCs	mesenchymal stem cells
OA	osteoarthritis
PT	physiotherapy
PRP	platelet-rich plasma
TKA	total knee arthroplasty
